# Anzi Tiaochong Tang inhibits trophoblast ferroptosis via the METTL14/m6A/SLC39A14 axis in recurrent spontaneous abortion

**DOI:** 10.3389/fphar.2026.1787584

**Published:** 2026-04-29

**Authors:** Suning Huang, Meng Yu, Yu Luo, Yan Chen, Shan He, Fei Ma, Yan Ning, Jiaman Wu

**Affiliations:** 1 Guangzhou University of Chinese Medicine, Guangzhou, Guangdong, China; 2 Department of Chinese Medicine, Shenzhen Maternity and Child Healthcare Hospital, Women and Children’s Medical Center, Southern Medical University, Shenzhen, Guangdong, China; 3 Shenzhen Key Laboratory of Maternal and Child Health and Diseases, Shenzhen, Guangdong, China; 4 Southern Medical University, Guangzhou, Guangdong, China

**Keywords:** Anzi Tiaochong Tang, ferroptosis, m6A methylation, METTL14, recurrent spontaneous abortion, SLC39A14

## Abstract

**Purpose:**

Recurrent spontaneous abortion (RSA) remains a major challenge in reproductive medicine, with unclear etiology in a substantial proportion of cases. Emerging evidence suggests that ferroptosis contributes to trophoblast dysfunction and may be involved in RSA pathogenesis. This study aimed to evaluate the therapeutic effects of Anzi Tiaochong Tang (AZTCT) on RSA and to elucidate its underlying molecular mechanisms, with a particular focus on ferroptosis.

**Methods:**

An RSA rat model was established using hydroxyurea and mifepristone to assess the therapeutic efficacy of AZTCT *in vivo*. For mechanistic investigation, ferroptosis was induced in human trophoblast HTR-8/SVneo cells using erastin. N6-methyladenosine methylation (m6A) was analyzed by bioinformatics, methylated RNA immunoprecipitation (MeRIP), and dual-luciferase reporter assays. Loss- and gain-of-function experiments, including METTL14 knockdown and SLC39A14 overexpression, were performed to validate the regulatory pathway.

**Results:**

AZTCT significantly improved pregnancy outcomes in RSA rats and enhanced trophoblast cell viability and migration while suppressing ferroptosis *in vitro*. Mechanistically, AZTCT upregulated METTL14 expression, which in turn increased m6A modification of SLC39A14 mRNA. This modification facilitated YTHDF2-dependent recognition, leading to reduced mRNA stability of SLC39A14. Functional rescue experiments further demonstrated that SLC39A14 overexpression reversed the protective effects mediated by METTL14.

**Conclusion:**

AZTCT alleviates RSA, at least in part, by inhibiting trophoblast ferroptosis through the METTL14/m6A/YTHDF2/SLC39A14 axis. These findings provide mechanistic insight into the therapeutic potential of AZTCT and identify a novel epigenetic target for RSA treatment.

## Introduction

Recurrent spontaneous abortion (RSA) is a common and complex complication of pregnancy, defined as the loss of three or more consecutive pregnancies before 28 weeks of gestation with the same partner (Wang et al., 2025). It affects approximately 5% of women of reproductive age (Li et al., 2021), and its etiology remains unclear in nearly 50% of cases (Zheng et al., 2024). With the increasing number of pregnancies at advanced maternal age, the incidence of RSA has risen in recent years (Song et al., 2019). RSA not only imposes a substantial psychological and physical burden on affected women but also represents a significant clinical and socioeconomic challenge. Current therapeutic strategies remain controversial due to the heterogeneity of the disease, the lack of specific clinical manifestations, and the limited understanding of its underlying mechanisms (Deng et al., 2022). Therefore, elucidating the pathogenesis of RSA and identifying effective therapeutic approaches are of critical importance.

In traditional Chinese medicine, RSA is primarily attributed to kidney deficiency, which is believed to impair reproductive function. Accordingly, therapeutic strategies focus on nourishing the kidney and promoting blood circulation to remove blood stasis. Kidney-tonifying and blood-activating herbal formulations have been widely applied in the clinical management of RSA, particularly in cases with unexplained etiology (Zhou et al., 2022). Among these, Anzi Tiaochong Tang (AZTCT), a refined herbal decoction developed and clinically applied at Shenzhen Maternity and Child Healthcare Hospital, has shown promising efficacy in improving pregnancy outcomes. Despite its clinical application, the molecular mechanisms underlying its therapeutic effects remain largely unclear.

Recent studies have highlighted the role of epigenetic regulation in pregnancy-related disorders. N6-methyladenosine (m6A) methylation is the most abundant internal modification of eukaryotic mRNA and is dynamically regulated by methyltransferases (“writers”), demethylases (“erasers”), and binding proteins (“readers”) (Wu et al., 2022). Dysregulation of m6A modification has been implicated in a wide range of pathological processes (Jiang et al., 2021). Notably, aberrant m6A methylation has been implicated in the pathogenesis of recurrent spontaneous abortion, with altered m6A modification patterns observed in decidual tissues from RSA patients (Luo et al., 2023). Methyltransferase-like 14 (METTL14), a core component of the m6A methyltransferase complex, plays a crucial role in RNA modification by forming a heterodimer with METTL3 (Liu et al., 2022). Previous studies have shown that METTL14 regulates trophoblast cell migration and invasion in unexplained RSA (Nong et al., 2025). However, its role in ferroptosis and its involvement in RSA pathogenesis remain to be fully elucidated.

Ferroptosis is a form of regulated cell death characterized by iron-dependent lipid peroxidation (Stockwell et al., 2017). Trophoblast cells are particularly susceptible to oxidative stress and iron overload, both of which are key drivers of ferroptosis (Beharier et al., 2021). Emerging evidence suggests that excessive ferroptosis contributes to trophoblast dysfunction, impaired placental development, and pregnancy-related disorders (Li et al., 2025). Notably, abnormal oxidative stress and dysregulated iron metabolism have been observed in RSA, indicating that ferroptosis may play a role in its pathogenesis (Gupta et al., 2007; Hussain et al., 2021). However, the molecular mechanisms linking ferroptosis to RSA remain largely unclear.

In this study, we employed a two-step experimental strategy to investigate the therapeutic effects and underlying mechanisms of AZTCT in RSA. First, an *in vivo* RSA rat model was established to evaluate the therapeutic efficacy of AZTCT. Second, an *in vitro* ferroptosis model using human trophoblast HTR-8/SVneo cells was constructed to explore the molecular mechanisms. We hypothesized that AZTCT alleviates RSA by regulating trophoblast ferroptosis through an m6A-dependent pathway. Our findings provide new insights into the epigenetic regulation of ferroptosis in RSA and offer a potential molecular basis for the clinical application of AZTCT.

## Materials and methods

### Composition and preparation of AZTCT

The pharmacological validation of AZTCT was performed in accordance with the Consortium for Phytochemical Methodology and Protocol guidelines for Type A extracts (Heinrich et al., 2022). AZTCT is a traditional Chinese medicine formulation composed of the following botanical materials: *Dipsaci Radix* (roots of *Dipsacus asper* Wall. ex Henry), *Spatholobi Caulis* (stems of *Spatholobus suberectus* Dunn), *Typhae Pollen Carbonisatus* (charred pollen of *Typha angustifolia* L.), *Ginseng Radix et Rhizoma* (roots and rhizomes of *Panax ginseng* C.A. Mey.), *Taxilli Herba* (stems and leaves of *Taxillus chinensis* DC. Danser), and *Citri Reticulatae Pericarpium* (pericarp of *Citrus reticulata* Blanco).

All botanical materials were purchased from Shenzhen Huahui Pharmaceutical Co., Ltd. (China) and authenticated by Professor Shan He (Shenzhen Maternity and Child Healthcare Hospital).

The crude drugs (total weight: 49 g) were soaked in distilled water (1:10, w/v) for 30 min and then refluxed for 30 min. The filtrate was collected, and the residue was extracted again with distilled water (1:5, w/v) for an additional 30 min. The combined filtrates were concentrated under reduced pressure at 60 °C and lyophilized to obtain a dried extract. The extraction yield was 14.8% (w/w), corresponding to a drug extract ratio of 6.7:1. The dried powder was stored at −20 °C and reconstituted in distilled water prior to use.

For *in vivo* administration, the extract was prepared at a concentration of 0.5145 g/mL (crude drug equivalent; 0.076 g/mL dried extract).

### Chemical profiling

The chemical composition of AZTCT was characterized by high-performance liquid chromatography–tandem mass spectrometry (HPLC–MS/MS) to identify quality markers (Q-markers). Major bioactive constituents, including liquiritigenin, nobiletin, ginsenoside Rb1, and astragalin, were quantitatively analyzed according to previously reported methods and patent standards (He et al., 2025).

### Animal study

Specific pathogen-free Sprague–Dawley rats (6 weeks old, 200–220 g) were obtained from SLAC Laboratory Animal Co., Ltd. (Shanghai, China). Animals were housed under controlled conditions (22 °C–25 °C, 12-h light/dark cycle) with free access to food and water. All procedures were conducted in accordance with the Guide for the Care and Use of Laboratory Animals.

After 1 week of acclimatization, rats were mated at a male:female ratio of 1:2. The presence of a vaginal plug or sperm in vaginal smears was defined as gestational day 0.

Pregnant rats were randomly assigned to four groups (n = 9 per group): model, saline, AZTCT, and positive control (PC). An RSA model was established using hydroxyurea and mifepristone based on an orthogonal experimental design according to previously described (Ding et al., 2024).

Rats in the model group received hydroxyurea (450 mg/kg/day) by intragastric administration from gestational day 1–9. The AZTCT group received AZTCT (5.145 g/kg/day crude drug equivalent) during the same period. The dose was calculated based on the clinical prescription (49 g/60 kg) using body surface area conversion. The PC group received dydrogesterone (3.02 mg/kg/day).

On gestational day 10, all rats were administered mifepristone (4 mg/kg). After 24 h, blood samples were collected, and serum was isolated by centrifugation. Decidual tissues were harvested for morphological and biochemical analyses. The abortion rate was calculated as the number of resorbed embryos divided by the total number of embryos.

### Hematoxylin and eosin (H&E) staining

Fresh decidual tissues were fixed in 10% neutral-buffered formalin for 24 h, embedded in paraffin, and sectioned at 4 μm thickness. Sections were deparaffinized, rehydrated, and stained with hematoxylin and eosin. Histopathological changes were examined under a light microscope.

### Radioimmunoassay

The concentrations of progesterone and estrone in serum were quantified using commercial radioimmunoassay kits (Gemic, Shanghai, China) according to the manufacturer’s protocols. All assays were performed in triplicate to ensure reproducibility.

### Enzyme-linked immunosorbent assay (ELISA)

Serum levels of vascular endothelial growth factor (VEGF) were determined using a rat VEGF ELISA kit (Beyotime, Shanghai, China). Antibodies against β2-glycoprotein I (β2-GP1) IgA, IgG, and IgM were quantified using a rat β2-GP1 IgA/G/M ELISA kit (Hengyuan Biotechnology, Shanghai, China). All procedures were conducted following the manufacturers’ instructions.

### Determination of superoxide dismutase (SOD) activity

Decidual tissues were homogenized in pre-cooled normal saline, followed by centrifugation at 10,000 × g for 15 min at 4 °C. The supernatant (10 μL) was mixed with 4.5 mL of Tris-HCl-EDTA solution (pH 8.2) at 25 °C, and SOD activity was measured spectrophotometrically at 325 nm.

### Quantitative real-time polymerase chain reaction (qPCR) analysis

Total RNA was extracted from decidual tissues using TRIzol reagent (Sangon, Shanghai, China). Complementary DNA (cDNA) synthesis was performed using the M-MuLV First Strand cDNA Synthesis Kit (Sangon). qPCR was carried out with 2X HyperMB Universal SYBR Green qPCR Master Mix (Sangon). Relative gene expression was calculated using the 2^−ΔΔCT^ method, with β-actin as the internal control. The primer sequences used for qPCR were listed in Supplementary Table S1.

### Cell culture and treatment

Human trophoblast HTR8-S/Vneo cells (ATCC, Manassas, VA, USA) were cultured in RPMI-1640 medium (Gibco, Grand Island, NY, USA) supplemented with 10% fetal bovine serum (FBS; Gibco) at 37 °C in a humidified atmosphere containing 5% CO_2_. For ferroptosis induction, cells were treated with 10 μM Erastin (MCE, Monmouth Junction, NJ, USA) for 24 h according to previously described (Yang et al., 2022). Subsequently, cells were treated with AZTCT extract at a final concentration of 1.6 mg/mL (dried extract equivalent) for 24 h to evaluate its effects on ferroptosis and cell migration. This concentration was selected based on previous studies investigating herbal extracts in trophoblast cells, where similar doses were shown to exert biological activity without significant cytotoxicity (He et al., 2025).

### Cell transfection

Cells were seeded in six-well plates to 80% confluence and transfected with shRNA targeting METTL14 (shMETTL14; sense: 5′-GAT​CCG​CTT​ACG​TTC​TCG​AAA​TTC​AAG​AGA​TTT​CGG​AAG​CAG​CTG​TTT​TTT-3′) cloned into pLKO.1-puro using Lipofectamine 2000 (Invitrogen, Carlsbad, CA, USA). Knockdown efficiency (∼82 ± 4%) was verified by Western blot (Supplementary Figure S1). Off-target effects were evaluated via BLAST to confirm specificity. METTL14 overexpression plasmids, SLC39A14 overexpression plasmids, shYTHDF2, and control plasmids (pcDNA3.1 or shNC) were transfected according to the manufacturer’s protocol. Cells were harvested 48 h post-transfection.

### Cell counting kit-8 (CCK-8) assay

Cells were seeded in 96-well plates and incubated for 24 h. CCK-8 reagent (10 μL; Beyotime) was added to each well and incubated for 4 h. Absorbance was measured at 450 nm using a microplate reader to assess cell viability.

### Transwell migration assay

Cell migration was evaluated using 24-well Transwell chambers (Corning, NY, USA). HTR8-S/Vneo cells were seeded in the upper chamber, and complete medium was added to the lower chamber. After 24 h, cells that migrated through the membrane were fixed with 4% paraformaldehyde and stained with crystal violet. Migrated cells were imaged under a light microscope.

### Detection of lipid reactive oxygen species (ROS)

Lipid ROS levels were measured using the C11 BODIPY 581/591 probe (Beyotime). Cells were washed with PBS, incubated with 1 mL of probe solution at 37 °C for 20 min, washed, and resuspended in PBS for fluorescence microscopy under UV illumination.

### Determination of intracellular Fe^2+^ concentration

Cellular Fe^2+^ levels were quantified using a ferrous iron colorimetric assay kit (Elabscience, Wuhan, China). Cells were lysed on ice for 10 min, centrifuged at 15,000 × g for 10 min, and 80 μL of supernatant was incubated with 80 μL of chromogenic solution at 37 °C for 10 min. Absorbance was measured at 593 nm.

### Glutathione (GSH) assay

Cells underwent two rapid freeze-thaw cycles using liquid nitrogen and a 37 °C water bath. After centrifugation at 10,000 × g for 10 min, the supernatant was incubated with protein removal reagent S and GSH detection working solution at 25 °C for 5 min. Following addition of 50 μL NADPH solution, absorbance was read at 412 nm.

### Malondialdehyde (MDA) assay

Lipid peroxidation was measured using an MDA assay kit (Beyotime). Cell lysates were incubated with MDA working solution at 100 °C for 15 min, cooled, centrifuged at 1,000 × g for 10 min, and absorbance was measured at 532 nm.

### TUNEL assay

Cell apoptosis was assessed using a one-step TUNEL assay kit (Beyotime). Cells were fixed with 4% paraformaldehyde, permeabilized with 0.3% Triton X-100, and incubated with TUNEL detection solution at 37 °C for 60 min. Nuclei were counterstained with 1 μg/mL DAPI (Sigma-Aldrich) for 10 min, and cells were observed using fluorescence microscopy.

### m6A quantification

Total m6A RNA methylation levels were measured using the EpiQuik m6A RNA methylation quantification kit (Epigentek, Farmingdale, NY, USA). Total RNA was extracted from HTR8-S/Vneo cells and incubated with binding buffer and anti-m6A antibody at 37 °C for 90 min, followed by capture and detection antibody incubations for 60 min and 30 min, respectively. Absorbance was measured at 450 nm after reaction with chromogenic substrate.

### Western blotting

Cells were lysed in RIPA buffer, and protein concentrations were determined using the BCA assay (Beyotime). Equal amounts of protein were separated by SDS-PAGE and transferred onto PVDF membranes. After blocking with 5% non-fat milk, membranes were incubated with primary antibodies against METTL14, METTL3, WTAP, RBM15, FTO, and ALKBH5 at 4 °C overnight, followed by incubation with appropriate secondary antibodies at room temperature for 1 h. Protein bands were visualized using enhanced chemiluminescence (ECL; Beyotime), with β-actin serving as the loading control.

### Bioinformatic analysis

Differentially expressed genes were obtained from the GSE165004 dataset downloaded from the Gene Expression Omnibus (GEO) database. To identify potential downstream targets of METTL14, correlation analysis between METTL14 and other genes was performed using the *cor* function in R. Pathway enrichment analysis of METTL14-correlated genes was performed using the Kyoto Encyclopedia of Genes and Genomes (KEGG) database to identify biologically relevant signaling pathways. Candidate target genes were further screened based on correlation strength and biological relevance.

### Methylated RNA immunoprecipitation (MeRIP)

m6A levels of SLC39A14 were determined using the m6A MeRIP kit (BersinBio, Guangzhou, China) following the manufacturer’s instructions. Total RNA was extracted from HTR8-S/Vneo cells and fragmented by ultrasonication and incubated with 4 μg of anti-m6A antibody at 4 °C for 4 h. Protein A/G magnetic beads were subsequently added and incubated at 4 °C for 1 h. After thorough washing and elution, immunoprecipitated RNA was isolated, and the levels of SLC39A14 were quantified using quantitative PCR (qPCR).

### Dual-luciferase reporter assay

Putative m6A modification sites in SLC39A14 were predicted using the SRAMP online tool. Wild-type (WT) and mutant (MUT) SLC39A14 sequences were cloned into pGL3-basic firefly luciferase reporter vectors (Promega, Madison, WI, USA). To evaluate the interaction between METTL14 and SLC39A14, HTR8-S/Vneo cells were co-transfected with METTL14 overexpression plasmids or pcDNA3.1 vector, WT or MUT reporter plasmids, and pRL-TK Renilla luciferase vectors (Promega) using Lipofectamine 2000. To assess the involvement of YTHDF2, similar co-transfections were performed using YTHDF2 overexpression plasmids or pcDNA3.1 vectors, together with WT or MUT reporter plasmids and pRL-TK vectors. Luciferase activity was measured 48 h post-transfection using the Dual-Luciferase Reporter Assay System (Promega).

### RNA stability assay

To evaluate the stability of SLC39A14 mRNA, HTR8-S/Vneo cells were treated with 2 μg/mL actinomycin D (Sigma-Aldrich) for 0, 4, 8, and 12 h to inhibit transcription. RNA was extracted at each time point, and SLC39A14 expression was quantified by qPCR.

### RNA immunoprecipitation (RIP)

The interaction between m6A reader proteins and SLC39A14 was assessed using the RIP kit (BersinBio). Cells were lysed in ice-cold lysis buffer containing protease inhibitors for 20 min, followed by centrifugation at 16,000 × g for 10 min. Supernatants were incubated with antibodies against YTHDF2, YTHDF3, YTHDC1, YTHDC2, or IgG control at 4 °C for 16 h, followed by incubation with Protein A/G magnetic beads for 1 h at 4 °C. After washing and elution, co-immunoprecipitated RNA was isolated, and SLC39A14 levels were quantified by qPCR.

### Statistical analysis

Data are presented as mean ± standard deviation (SD). Statistical analyses were performed using GraphPad Prism 8. Differences between groups were analyzed using Student’s t-test or one-way ANOVA. A p-value <0.05 was considered statistically significant.

## Results

### Optimization of RSA rat model

An orthogonal experimental design was employed to determine the optimal concentrations of hydroxyurea and mifepristone for establishing the RSA rat model. As summarized in Supplementary Table S2, hydroxyurea and mifepristone were tested at multiple dosage levels, and the corresponding combinations were applied according to the orthogonal table (Supplementary Table S3). The abortion rate, embryo diameter, and serum levels of progesterone and estrone were subsequently assessed.

The abortion rate was highest in model 5 and lowest in model 4 (Supplementary Figure S2A). Embryo diameters were largest in model 1, followed by model 4, and shortest in model 5 (Supplementary Figure S2B). Serum progesterone and estrone levels were also highest in model 1 and lowest in model 5 (Supplementary Figures S2C,D). Based on these parameters, model 5, comprising 450 mg/kg hydroxyurea and 4 mg/kg mifepristone, was selected as the optimal RSA model for subsequent experiments.

### AZTCT improves RSA in rats

The effects of AZTCT on RSA were evaluated using the established rat model. The negative control group received normal saline, while the positive control (PC) group was administered dydrogesterone.

**FIGURE 1:**
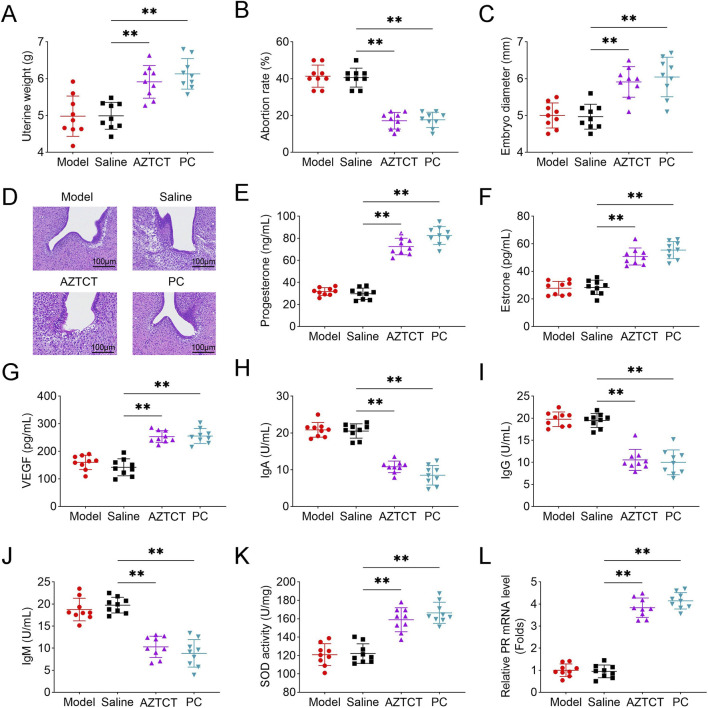
AZTCT ameliorates recurrent spontaneous abortion (RSA) in rats. RSA was induced in rats via intraperitoneal administration of 450 mg/kg hydroxyurea and 4 mg/kg mifepristone. Rats were subsequently treated with normal saline (saline, negative control), AZTCT (treatment), or dydrogesterone (positive control, PC) via intragastric administration. **(A)** Uterine weight. **(B)** Abortion rate. **(C)** Embryo diameter. **(D)** Histopathological evaluation of decidual tissues by H&E staining. Serum levels of **(E)** progesterone and **(F)** estrone were measured by radioimmunoassay. **(G)** Serum VEGF was quantified using ELISA. Levels of **(H)** β2-GP1 IgA, **(I)** IgG, and **(J)** IgM were assessed by β2-GP1 ELISA. **(K)** SOD activity in decidual tissues was measured using the pyrogallol autoxidation method. **(L)** Progesterone receptor (PR) mRNA expression in decidual tissues was assessed by qPCR. Data are presented as mean ± SD (n = 9). **P < 0.01 vs. saline group.

Compared with the saline group, uterine weight was significantly increased in both the AZTCT and PC groups (Figure 1A). The abortion rate was significantly reduced following AZTCT or dydrogesterone treatment (Figure 1B), and the mean embryo diameter was significantly larger in these groups relative to saline controls (Figure 1C). Histological analysis of decidual tissues revealed disrupted architecture and irregular glandular arrangement in the model and saline groups, whereas AZTCT or dydrogesterone administration restored tissue structure and glandular organization (Figure 1D).

Serum progesterone and estrone levels were significantly elevated in the AZTCT and PC groups compared with saline controls (Figures 1E,F). In addition, AZTCT treatment significantly increased serum vascular endothelial growth factor levels (Figure 1G), while reducing β2-glycoprotein 1 (β2-GP1) IgA, IgG, and IgM concentrations (Figures 1H–J). Moreover, AZTCT administration markedly enhanced superoxide dismutase activity and progesterone receptor (PR) mRNA expression in decidual tissues (Figures 1K,L).

Collectively, these results indicate that AZTCT effectively improves decidual morphology, hormonal profiles, and antioxidative capacity, thereby ameliorating RSA in this rat model.

### AZTCT promotes migration and inhibits ferroptosis in trophoblast cells

**FIGURE 2:**
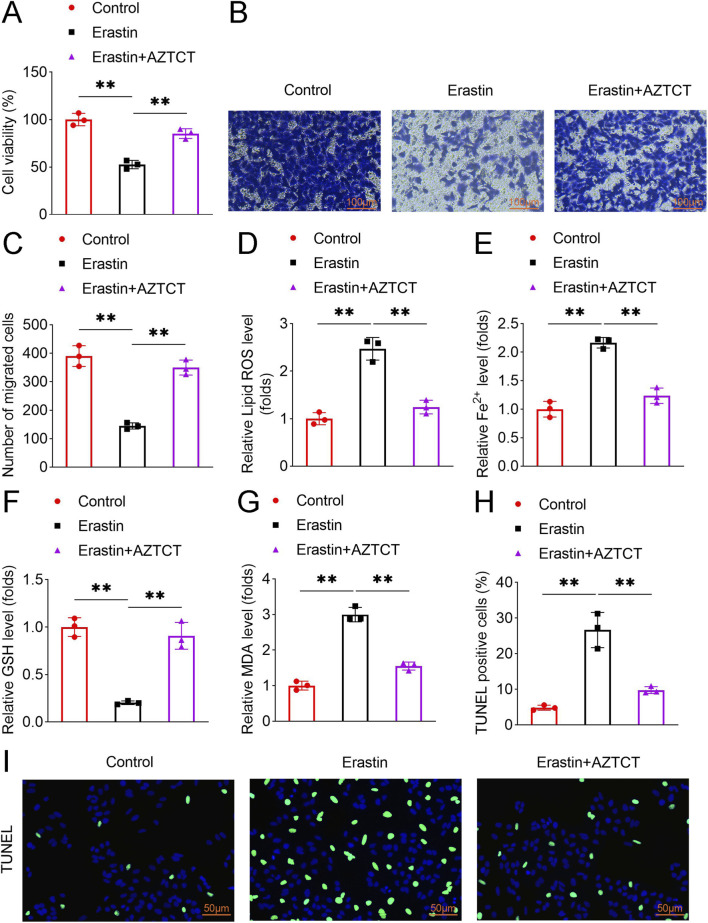
AZTCT promotes migration and inhibits ferroptosis in trophoblast cells. HTR8-S/Vneo cells were treated with Erastin with or without AZTCT. **(A)** Cell viability was assessed by CCK-8 assay. **(B,C)** Cell migration was evaluated using Transwell assay, and the number of migrated cells was quantified. **(D)** Lipid ROS levels were measured using the C11 BODIPY 581/591 probe. **(E–G)** Fe^2+^, GSH, and MDA levels were quantified using commercial assay kits. **(H,I)** Cell apoptosis was assessed by TUNEL assay, with representative images shown. Data are presented as mean ± SD (n = 3). **P < 0.01.

To investigate the mechanistic effects of AZTCT on trophoblast cell behavior, HTR8-S/Vneo cells were treated with Erastin to induce ferroptosis, followed by AZTCT administration. Erastin significantly reduced cell viability, whereas AZTCT partially restored cell viability (Figure 2A). Similarly, Erastin inhibited cell migration, which was reversed by AZTCT treatment (Figures 2B,C).

Assessment of ferroptosis-related markers revealed that Erastin increased lipid reactive oxygen species (ROS), Fe^2+^, and malondialdehyde (MDA) levels, while reducing glutathione (GSH) levels. AZTCT counteracted these effects, restoring redox balance (Figures 2D–G). TUNEL assays further confirmed that Erastin induced cell death, which was significantly attenuated by AZTCT (Figures 2H,I). Collectively, these results indicate that AZTCT enhances trophoblast migration and mitigates Erastin-induced ferroptosis.

### AZTCT enhances METTL14-mediated m6A methylation

**FIGURE 3:**
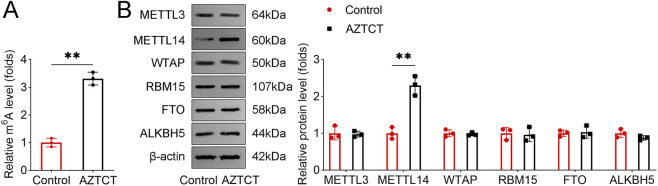
AZTCT promotes METTL14-mediated m6A methylation. **(A)** Total m6A levels in HTR8-S/Vneo cells after AZTCT treatment were quantified using an m6A RNA methylation kit. **(B)** Protein levels of METTL3, METTL14, WTAP, RBM15, FTO, and ALKBH5 were analyzed by Western blotting. Data are presented as mean ± SD (n = 3). **P < 0.01.

Given the role of N6-methyladenosine (m6A) in RNA regulation, we examined whether AZTCT modulates m6A methylation. AZTCT treatment increased global m6A levels in HTR8-S/Vneo cells (Figure 3A). Western blot analysis showed that AZTCT specifically upregulated METTL14 protein expression, while the levels of METTL3, WTAP, RBM15, FTO, and ALKBH5 remained unchanged (Figure 3B). These findings suggest that AZTCT promotes m6A methylation in trophoblast cells predominantly through METTL14.

### METTL14 mediates the effects of AZTCT on trophoblast cell migration and ferroptosis

**FIGURE 4:**
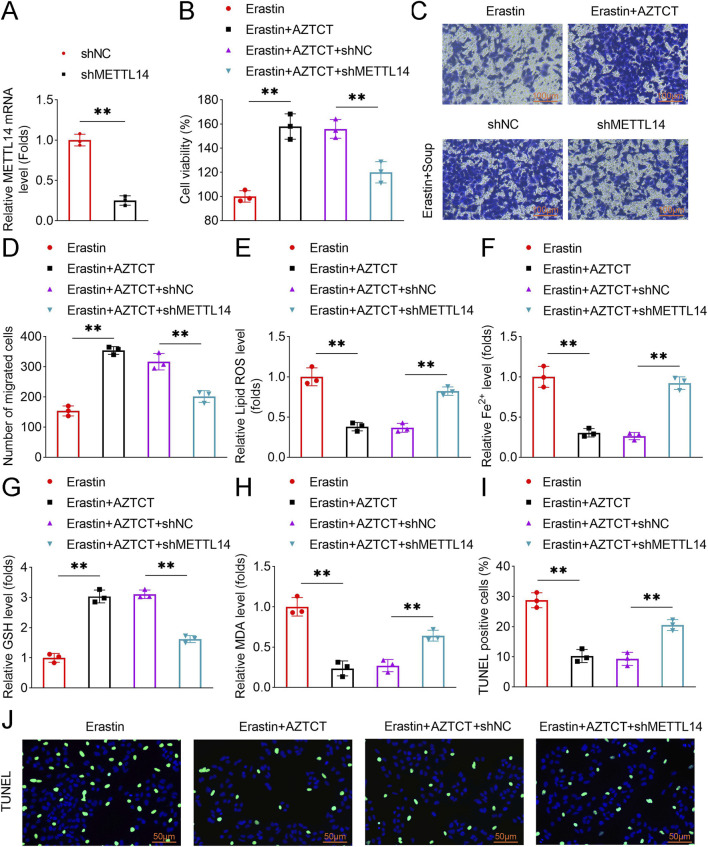
AZTCT enhances trophoblast cell migration and inhibits ferroptosis via METTL14. HTR8-S/Vneo cells were transfected with shMETTL14 and treated with Erastin and AZTCT. **(A)** METTL14 expression was verified by qPCR. **(B)** Cell viability was assessed by CCK-8. **(C,D)** Cell migration was evaluated by Transwell assay and quantified. **(E–H)** Lipid ROS, Fe^2+^, GSH, and MDA levels were measured. **(I,J)** Apoptosis was assessed by TUNEL assay, with representative images shown. Data are presented as mean ± SD (n = 3). **P < 0.01.

To determine the functional significance of METTL14, rescue experiments were conducted using shRNA-mediated knockdown of METTL14 (shMETTL14) in HTR8-S/Vneo cells (Figure 4A). AZTCT-induced enhancement of cell viability and migration was abolished upon METTL14 knockdown (Figures 4B–D). Similarly, AZTCT-mediated reductions in lipid ROS, Fe^2+^, MDA, and TUNEL-positive cells, as well as the increase in GSH levels, were reversed by METTL14 knockdown (Figures 4E–J). These results indicate that METTL14 is required for AZTCT-mediated promotion of migration and inhibition of ferroptosis.

### METTL14 reduces SLC39A14 mRNA stability via m6A methylation

**FIGURE 5:**
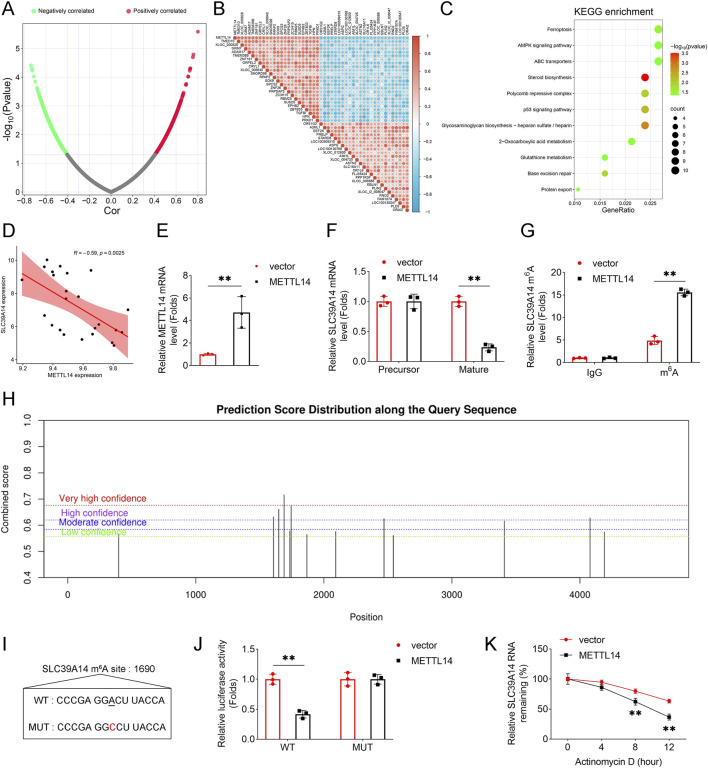
METTL14 reduces SLC39A14 mRNA stability via m6A methylation. **(A,B)** Genes positively and negatively correlated with METTL14 were identified. **(C)** KEGG pathway enrichment analysis of METTL14-correlated genes. **(D)** Correlation between METTL14 and SLC39A14 expression. **(E)** METTL14 overexpression was confirmed by qPCR. **(F)** Expression of precursor and mature SLC39A14 mRNA after METTL14 overexpression. **(G)** m6A levels of SLC39A14 were measured by MeRIP. **(H,I)** Predicted m6A sites and motif in SLC39A14. **(J)** Binding of METTL14 to SLC39A14 m6A site was validated by dual-luciferase reporter assay. **(K)** SLC39A14 mRNA stability was assessed after actinomycin D treatment. Data are presented as mean ± SD (n = 3). **P < 0.01.

To identify downstream targets of METTL14, gene correlation analysis was performed, revealing a set of positively and negatively correlated genes enriched in ferroptosis-related pathways (Figures 5A–C). Among these, SLC39A14 was negatively correlated with METTL14 expression (R = −0.59, P = 0.0025; Figure 5D).

METTL14 overexpression selectively reduced mature SLC39A14 mRNA levels without affecting its precursor transcript (Figures 5E,F), and increased m6A modification on SLC39A14 mRNA (Figure 5G). Bioinformatic prediction identified multiple potential m6A sites (Figure 5H), and the site at position 1690 was experimentally validated (Figures 5I,J). Actinomycin D assays demonstrated that METTL14 decreased SLC39A14 mRNA stability (Figure 5K), indicating that METTL14 promotes m6A modification of SLC39A14, leading to transcript destabilization.

### METTL14/SLC39A14 axis regulates trophoblast cell migration and ferroptosis

**FIGURE 6:**
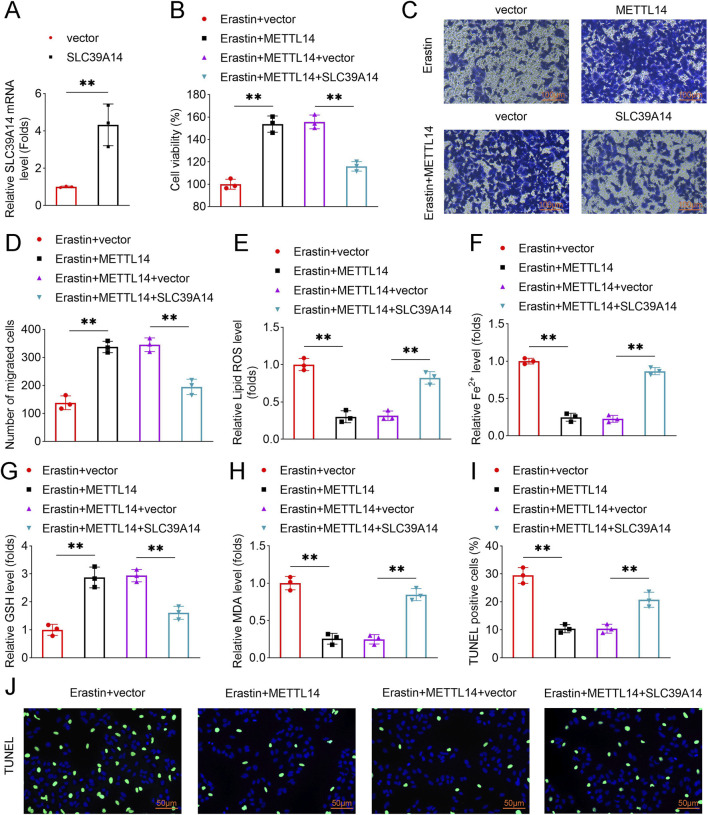
METTL14/SLC39A14 axis regulates trophoblast migration and ferroptosis. HTR8-S/Vneo cells were co-transfected with METTL14 and SLC39A14 overexpression plasmids and treated with Erastin. **(A)** SLC39A14 expression was measured by qPCR. **(B)** Cell viability was assessed by CCK-8. **(C, D)** Cell migration was evaluated using Transwell assay. **(E–H)** Lipid ROS, Fe^2+^, GSH, and MDA levels were measured. **(I,J)** Cell apoptosis was quantified by TUNEL assay, with representative images shown. Data are presented as mean ± SD (n = 3). **P < 0.01.

Rescue experiments were performed to examine the role of SLC39A14 in METTL14-mediated cellular effects. SLC39A14 overexpression attenuated METTL14-induced increases in cell viability and migration (Figures 6A–D). Similarly, METTL14-mediated reductions in lipid ROS, Fe^2+^, MDA, and TUNEL-positive cells, as well as increases in GSH levels, were reversed by SLC39A14 overexpression (Figures 6E–J). These results demonstrate that METTL14 promotes trophoblast migration and inhibits ferroptosis by downregulating SLC39A14.

### YTHDF2 recognizes METTL14-mediated m6A methylation of SLC39A14

**FIGURE 7:**
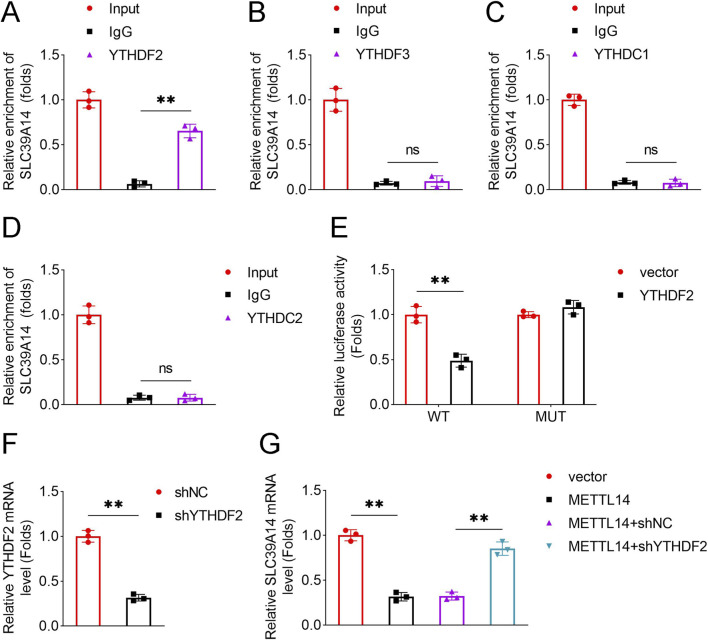
YTHDF2 recognizes METTL14-mediated m6A modification of SLC39A14. RIP assay was performed to detect interaction between SLC39A14 and **(A)** YTHDF2, **(B)** YTHDF3, **(C)** YTHDC1, or **(D)** YTHDC2. **(E)** Dual-luciferase reporter assay confirmed binding of YTHDF2 to the SLC39A14 m6A site. **(F)** YTHDF2 knockdown efficiency was validated by qPCR. **(G)** Effects of METTL14 overexpression and YTHDF2 knockdown on SLC39A14 expression were assessed by qPCR. Data are presented as mean ± SD (n = 3). **P < 0.01; ns, not significant.

To identify the m6A “reader” responsible for recognizing SLC39A14, RIP assays were performed with YTHDF2, YTHDF3, YTHDC1, and YTHDC2. Only YTHDF2 exhibited a significant interaction with SLC39A14 (Figures 7A–D). Dual-luciferase reporter assays confirmed that YTHDF2 decreased luciferase activity of the SLC39A14 wild-type construct but not the mutant construct (Figure 7E). Knockdown of YTHDF2 restored SLC39A14 expression reduced by METTL14 overexpression (Figures 7F,G), indicating that YTHDF2 mediates recognition and downstream effects of METTL14-induced m6A modification on SLC39A14.

## Discussion

Previous studies by our group have elucidated aspects of the mechanism of action of Anzi Tiaochong Tang in recurrent spontaneous abortion (Ma et al., 2021; Ma et al., 2024; He et al., 2025). Building upon this foundation, the present study further delineates the molecular pathways through which AZTCT exerts its therapeutic effects.

Initial *in vivo* experiments demonstrated that AZTCT significantly reduced miscarriage rates and improved uterine health in RSA rats. These phenotypic improvements were accompanied by elevated serum progesterone and estrone levels, as well as increased expression of progesterone receptor in decidual tissues, suggesting that AZTCT may rescue luteal phase defects, a well-recognized contributor to RSA pathogenesis (Kratz et al., 2017). Moreover, AZTCT increased serum vascular endothelial growth factor levels, indicating enhanced angiogenesis at the maternal-fetal interface, which is critical for placental development and nutrient supply (Wang et al., 2020). Autoimmune factors, particularly antiphospholipid antibodies, are known to impair fertility and are strongly associated with RSA (Cervera and Balasch, 2008). Notably, AZTCT administration reduced serum β2-glycoprotein I, IgA, IgG, and IgM levels, suggesting that AZTCT may mitigate the autoimmune component of RSA. In addition, AZTCT enhanced superoxide dismutase activity in decidual tissues, potentially alleviating oxidative damage and improving placental vascular function and immune homeostasis (Gupta et al., 2007). Collectively, these findings indicate that AZTCT exerts multifaceted protective effects in RSA.

Trophoblast cells are the primary functional units of the developing placenta, and their impaired migration or survival underlies many pregnancy complications (Xiao et al., 2020). Migration of trophoblasts is essential for proper placentation (Silva and Serakides, 2016), and abnormal trophoblast cell death is strongly associated with impaired migration (Chen et al., 2023). Ferroptosis, a form of regulated cell death characterized by iron-dependent lipid peroxidation, occurs preferentially in trophoblast cells compared with endometrial or decidual cells (Ng et al., 2019). Dysregulated ferroptosis has been implicated in the pathogenesis of RSA (Qi et al., 2022; Li et al., 2024). In the present study, AZTCT inhibited ferroptosis and promoted trophoblast migration *in vitro*, suggesting that its beneficial effects on placental physiology contribute to the reduction in miscarriage rates observed *in vivo*.

N6-methyladenosine modification has been reported to influence cellular susceptibility to ferroptosis (Shi et al., 2024). Here, AZTCT treatment increased METTL14-mediated m6A methylation in trophoblast cells. METTL14 has previously been reported to regulate trophoblast migration and invasion in RSA (Nong et al., 2025). However, its role in ferroptosis remained unexplored. Our results demonstrate that METTL14 knockdown reversed AZTCT-induced promotion of migration and inhibition of ferroptosis, indicating that AZTCT attenuates RSA, at least in part, by modulating METTL14-dependent trophoblast ferroptosis.

Mechanistically, METTL14 was found to promote m6A methylation of SLC39A14, an iron transporter, leading to reduced mRNA stability, a process recognized by the m6A “reader” YTHDF2. This represents a novel epigenetic mechanism by which AZTCT regulates iron homeostasis in trophoblasts. While SLC39A14 is known to mediate non-transferrin-bound iron uptake (Liuzzi et al., 2006), its functional role in RSA had not been characterized. *In vitro* experiments demonstrated that SLC39A14 overexpression counteracted the METTL14-mediated promotion of migration and inhibition of ferroptosis, confirming its functional significance. Collectively, these findings position AZTCT as a regulator of the METTL14-m6A-YTHDF2-SLC39A14 axis, providing a potential molecular target for therapeutic intervention in RSA.

Nevertheless, several limitations should be acknowledged. First, the mechanistic findings were primarily derived from *in vitro* experiments using human trophoblast cells; the causal relationships *in vivo* have not been fully validated. Second, while the rat RSA model recapitulates some pathological features of human RSA, interspecies differences may limit the direct extrapolation of these findings to clinical settings. Third, clinical evidence in RSA patients is currently lacking, and further studies involving patient-derived tissues or clinical trials are necessary to confirm the translational relevance of the METTL14-m6A-SLC39A14 axis and the therapeutic potential of AZTCT.

## Conclusion

This study demonstrates that AZTCT exerts therapeutic effects in RSA by inhibiting trophoblast ferroptosis and promoting cell migration via the METTL14/m6A/SLC39A14 axis in a YTHDF2-dependent manner. These findings highlight the potential of AZTCT as a treatment for RSA and suggest that targeting m6A methylation may represent a novel therapeutic strategy. However, the mechanistic evidence is primarily derived from *in vitro* studies, and further *in vivo* and clinical investigations are needed to validate these effects.

## Data Availability

The data that support the findings of this study are available on request from the corresponding authors, upon reasonable request.
