# Supplementary material for: Anzi Tiaochong Tang inhibits trophoblast ferroptosis via the METTL14/m6A/SLC39A14 axis in recurrent spontaneous abortion
Source: Front Pharmacol. 2026 Apr 29;17:1787584. doi: 10.3389/fphar.2026.1787584 (PMC13167929; doi:10.3389/fphar.2026.1787584)
Supplement: Supplementary file 1 [file Supplementaryfile1.pdf]

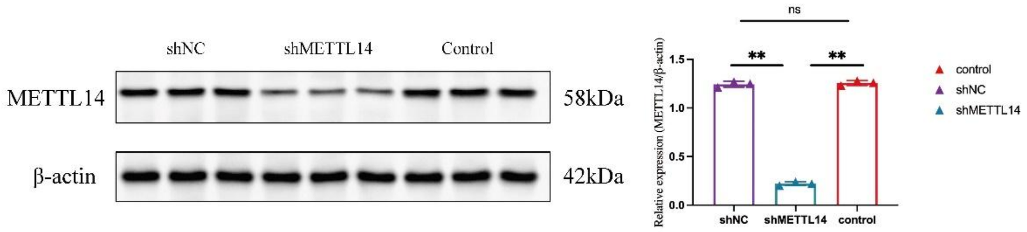

Supplementary Figure 1. Validation of METTL14 knockdown efficiency using Western blot.

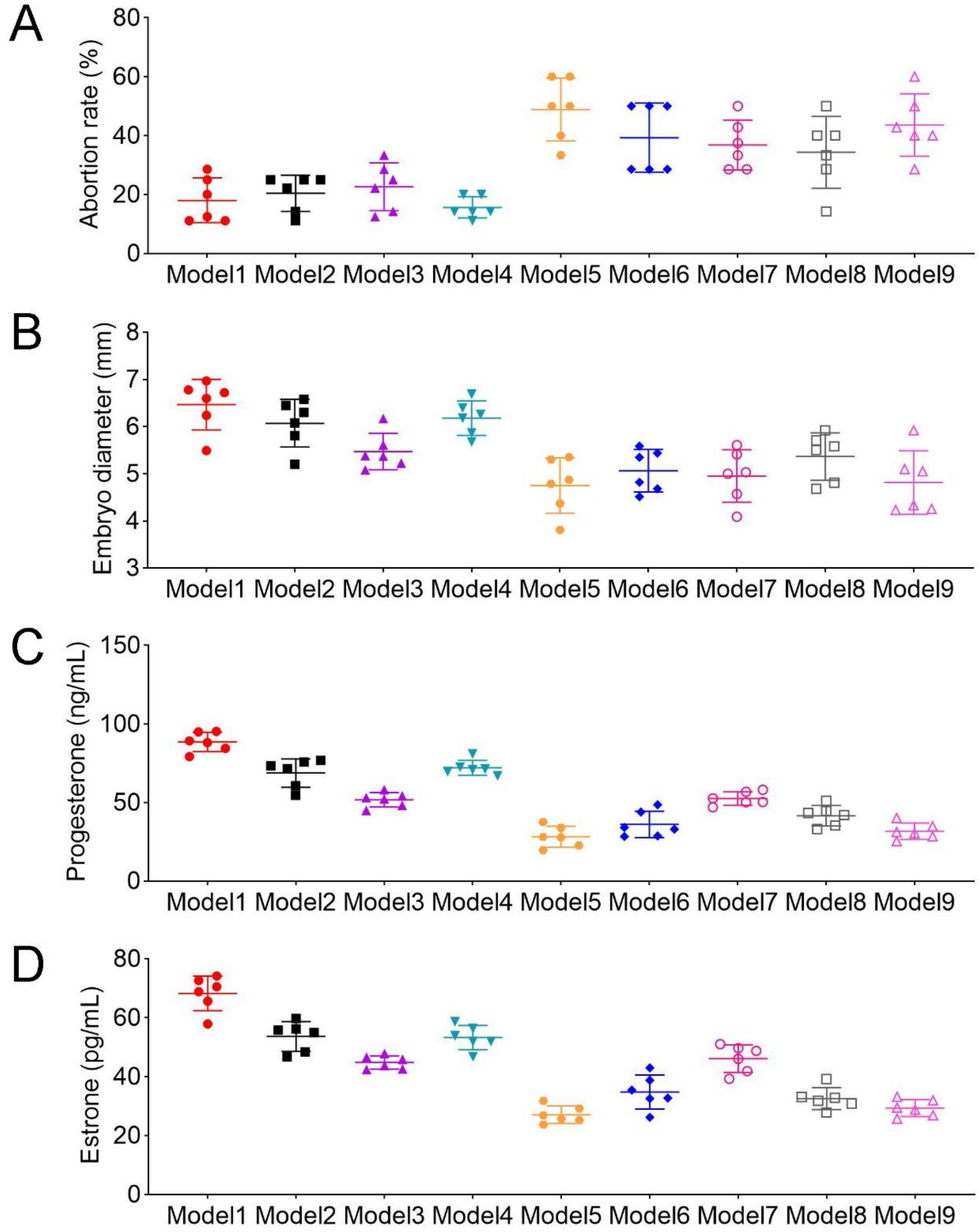

Supplementary Figure 2. RSA rat model optimization. RSA models were established according to an orthogonal design, and the following indicators were measured: (A) abortion rate, (B) embryo diameter, (C) serum progesterone, and (D) serum estrone. Data are presented as mean ± SD (n = 6).

Supplementary Table S1. Primer sequences used for qPCR

Gene	Species	Forward (5′–3′)	Reverse (5′–3′)
SLC39A14	Human	GAGGCTCACGCTTCATCCC	CCCTCGCCATACCGATGTATTA
METTL14	Human	AGTGCCGACAGCATTGGTG	GGAGCAGAGGTATCATAGGAAGC
YTHDF2	Human	AGCCCCACTTCCTACCAGATG	TGAGAACTGTTATTTCCCCATGC
β-actin	Human	CATGTACGTTGCTATCCAGGC	CTCCTTAATGTCACGCACGAT
PR	Rat	CTTCCCAGACTGCACCTACC	CCATATTGTGGGCTCTGGCT
β-actin	Rat	CCCGCGAGTACAACCTTCTT	AACACAGCCTGGATGGCTAC

Supplementary Table S2. Factor and level table

Factor		A:Hydroxyurea	B:Mifepristone
Level	1	400mg/kg	3mg/kg
	2	450mg/kg	4mg/kg
	3	500mg/kg	5mg/kg

Supplementary Table S3. Orthogonal table of hydroxyurea and mifepristone for $L_9(3^4)$

Group	Contribution of A Hydroxyurea	Contribution of B Mifepristone	Contribution degree of the interaction between A and B	System error detection
Model 1	1	1	1	1
Model 2	1	2	2	2
Model 3	1	3	3	3
Model 4	2	1	2	3
Model 5	2	2	3	1
Model 6	2	3	1	2
Model 7	3	1	3	2
Model 8	3	2	1	3
Model 9	3	3	2	1
